# Dimensions and correlates of quality of life according to frailty status: a cross-sectional study on community-dwelling older adults referred to an outpatient geriatric service in Italy

**DOI:** 10.1186/1477-7525-8-56

**Published:** 2010-06-08

**Authors:** Claudio Bilotta, Ann Bowling, Alessandra Casè, Paola Nicolini, Sabrina Mauri, Manuela Castelli, Carlo Vergani

**Affiliations:** 1 Department of Internal Medicine, Geriatric Medicine Unit, Fondazione IRCCS Cà Granda Ospedale Maggiore Policlinico, University of Milan, Milan, Italy; 2 Department of Primary Care and Population Health, University College London, Hampstead Campus, London, UK

## Abstract

**Background:**

There is a lack of knowledge concerning the relationship between two closely-linked multidimensional variables: frailty and quality of life (QOL). The aim of this study was to investigate dimensions and correlates of QOL associated with frailty status among community-dwelling older outpatients.

**Methods:**

We conducted a cross-sectional survey of 239 community-dwelling outpatients aged 65+ (mean age 81.5 years) consecutively referred to a geriatric medicine clinic in Italy between June and November 2009. Participants underwent a comprehensive geriatric assessment, including assessment of their frailty status according to the Study of Osteoporotic Fractures (SOF) criteria, and QOL, which was evaluated by using the Older People's QOL (OPQOL) questionnaire. One-way ANOVA and chi-squared tests were used to find correlates of frailty, including QOL dimensions, after stratification of participants in the "robust" (*n *= 72), "pre-frail" (*n *= 89) and "frail" (*n *= 78) groups. Multiple linear regression analyses were performed to find correlates of QOL in the overall sample and among "frail" and "robust" participants.

**Results:**

A negative trend of QOL with frailty status was found for almost all dimensions of QOL (health, independence, home and neighbourhood, psychological and emotional well-being, and leisure, activities and religion) except for social relationships and participation and financial circumstances. Independent correlates of a poor QOL in the total sample were "reduced energy level" (SOF criterion for frailty), depressive status, dependence in transferring and bathing abilities and money management (adjusted R squared 0.39); among "frail" participants the associations were with depressive status and younger age, and among "robust" participants the association was with lower body mass index.

**Conclusions:**

Five out of seven dimensions of QOL were negatively affected by frailty, but only one SOF criterion for frailty was independently related to QOL, after correction for age, functional status and depression. A more advanced age as well as a better affective status were correlates of a better QOL among frail elders. Interventions targeting the QOL in frail community-dwelling older outpatients should consider as outcomes, not only health-related QOL, but also other domains of the QOL.

## Background

Frailty has been defined as a state of increased vulnerability to stressors that results from decreased physiological reserves, and even dysregulation, of multiple physiologic systems [1]. Whether it be considered a *state variable *resulting from the accumulation of deficits [2] or a specific *clinical phenotype*, separate but partly overlapping with the concepts of chronic disease and disability [1,3], frailty is a well-known risk factor for adverse events such as functional decline, hospitalisation and death [1-3] and it has recently been shown to represent the main cause of death among community-dwelling older people [4]. Moreover, frailty has been associated with a significant impairment in the quality of life (QOL) [1-3]. QOL has been defined as an individual's perception of their position in life in the context of the culture and value system in which they live and in relation to their goals, expectations, standards and concerns [5]. It is a multidimensional variable and its correlates may be different according to the specific contexts [6,7].

Only very few randomised controlled trials targeting frail older people have considered QOL among outcomes, and QOL has seldom been shown to be improved [8,9]. Furthermore, many studies on frailty have taken into account the self-perceived health status or the health-related QOL and have not assessed QOL in general [3,10-14]. Masel and colleagues recently reported that in older Mexican Americans being pre-frail or frail was associated with worse scores on all physical and mental health-related quality of life scales than being non-frail [13]. However, they were unable to examine possible associations between frailty status and the social context of QOL because the QOL measures used in their research were health-focused.

More generally, it has been proposed that the onset of frailty is associated with an identity crisis, the so-called *frailty identity crisis*, a psychological syndrome that may accompany the transition from robustness to the "next to last" stage of life [15]. The psychological challenges stemming from the development of frailty such as regrets, sadness and depression can complicate physical frailty itself and have received little attention in literature so far; therefore, the need for studies on the independent correlates and outcomes of the *frailty identity crisis*, including QOL, has been recently highlighted [15].

Thus little is still known on the relationship between frailty status and the different aspects of QOL in general as well as on the interventions to improve QOL in frail elders. The aims of this study were i) to find out which dimensions of QOL are associated with frailty status among community-dwelling older outpatients referred to a geriatric medicine clinic in Italy, and ii) to investigate independent correlates of QOL both in the overall sample and in two specific subgroups, the frail and robust older adults.

## Methods

### Design, setting and participants

This observational cross-sectional study has considered 302 community-dwelling outpatients aged 65+ who consecutively underwent a first geriatric visit at the Fondazione Cà Granda Ospedale Maggiore Policlinico in Milan, Italy, from June 15 to November 15 2009. All subjects had been referred to this outpatient clinic by their general practitioners. All patients underwent a comprehensive geriatric assessment (CGA), which constitutes a standard procedure of the visit and includes both an evaluation of cognitive status by means of the Mini-Mental State Examination (MMSE) [16] and an evaluation of frailty status according to the recent Study of Osteoporotic Fractures (SOF) criteria [10,17]. Study participants were asked to fill in a general questionnaire on the QOL, called Older People's Quality of Life (OPQOL) [18-20], which is described below. The compilation of the questionnaire was carried out in the waiting room, before the visit; it was done by the patient completely on his/her own or with the help of a non-health volunteer who had been trained for the task, i.e. had been instructed to read out the questions, explain them when required and/or note down the answers chosen by the participant. If an informal caregiver accompanied the patient he/she was invited to refrain from influencing the choice of the answer, which had to be made by the older participant him/herself.

In order to ensure that the answers to the OPQOL would be reliable we excluded from the study subjects with severe cognitive impairment, indicated by a MMSE score < 11 out of 30 (*n *= 20) [21-23]. We also excluded from the study subjects who were unable to fill in the questionnaire properly because they did not understand all the questions (*n *= 26), those who refused to answer the questionnaire (*n *= 12) and those who did not give their written informed consent to the study (*n *= 5). The study therefore enrolled a sample of 239 community-dwelling older outpatients.

### Comprehensive geriatric assessment

The CGA included the main demographic, social and environmental characteristics of the participants, the occurrence of specific life events in the year prior to the visit, functional and physical status, comorbidity and frailty status. It was carried out during the visit by a multi-professional team which included a geriatrician and a professional nurse. The demographic characteristics considered were: age, gender, years of schooling and civil status. A number of social and environmental characteristics were also taken into account: living alone, home ownership status, home surface area, yearly family income, main characteristics of the carers, both informal and formal (if present). We also considered the occurrence of specific life events in the year prior to the visit: bereavement of partner or other family member, falls, admittance to the emergency department, hospitalisation, diseases with a severe prognosis (such as pneumonia, myocardial infarction, stroke, hypokinetic syndrome due to a bone fracture) and being victim of crime.

Functional status was assessed by means of the scales for the Basic Activities of Daily Living (BADL) [24] and the Instrumental Activities of Daily Living (IADL) [25], cognitive status by means of the MMSE scale with score correction for age and education [16], severity of dementia by means of the Clinical Dementia Rating (CDR) scale [26], emotional status by means of the 30-item Geriatric Depression Scale (GDS) [27] in subjects without dementia and with mild dementia (i.e. CDR score below 2 out of 5) and the Cornell scale for depression in dementia in the remaining subjects [28]. Comorbidity was assessed by means of the Cumulative Illness Rating Scale morbidity (CIRS-m) scale [29] and by considering any osteomuscular disease - given the correlation between this variable and the health-related QOL [13] - and the number of drugs taken daily. The diagnoses of dementia and depression were made according to the criteria of the Diagnostic and Statistical Manual of Mental Disorders fourth edition text revision (DSM-IV-TR) [30]. Weight, height and body mass index (BMI) (the weight in kilograms divided by the square of the height in metres) of participants were all measured during the visit with patients wearing light clothing, without shoes.

### Frailty status and QOL assessment

The frailty status of the participants was evaluated according to the recent Study of Osteoporotic Fractures (SOF) criteria, which are regarded to be just as effective as the frailty criteria of Fried *et al*. [3] in predicting adverse health outcomes but are easier to apply [10,17,31]. The SOF index is composed of three items: 1) intentional or unintentional weight loss > 5% in the past year, 2) inability to rise from a chair five consecutive times without using the arms, 3) self-perceived reduced energy level as described by a negative answer to the question "*do you feel full of energy?*". Subjects are considered "frail" if at least two of the three criteria are fulfilled, "pre-frail" if only one criterion is present and "robust" if none of the criteria are present.

QOL of the participants was evaluated by means of the OPQOL questionnaire, which has been recently validated on a community-dwelling older population in England [18-20]. It consists of 35 statements with the participant being asked to indicate the extent to which he/she agrees with every single statement by choosing one of five possible options among "strongly disagree", "disagree", "neither agree nor disagree", "agree" and "strongly agree". Each of the five possible answers is given a score of 1 to 5 so that higher scores indicate a better QOL. Thus the total score ranges from 35 (the worst possible QOL) to 175 (the best possible QOL). The 35 statements of the questionnaire consider the following aspects of QOL: life overall (score range 4-20), health (4-20), social relationships and participation (5-25), independence, control over life and freedom (4-20), home and neighbourhood (4-20), psychological and emotional well-being (4-20), financial circumstances (4-20), leisure, activities and religion (6-30).

### Statistical analyses and sample size calculations

In order to find out which dimensions of QOL were associated with the frailty syndrome participants were stratified into three groups, namely the "robust", "pre-frail" and "frail" groups according to the SOF criteria. The one-way ANOVA for metric variables with a normal distribution and Pearson's chi-squared test or Fisher's exact test for nominal variables were used in order to verify the null-hypothesis that the different dimensions of QOL as described by OPQOL sub-scores, as well as the characteristics of participants including the OPQOL total score, were similar across the three above-mentioned groups.

In order to investigate the characteristics associated with QOL in the sample overall as well as in the "frail" and in the "robust" groups, participants were stratified into three groups according to the lowest, intermediate and highest tertiles of the OPQOL total score. The one-way ANOVA for metric variables with a normal distribution and Pearson's chi-squared test or Fisher's exact test for nominal variables were also used in order to verify the null-hypothesis that the characteristics of the older participants were similar in the three QOL-related groups. A *P*-value less than or equal to 0.05 was assumed to indicate statistical significance.

As far as multivariate analyses in the total sample were concerned, two models of linear regression analysis were developed, both assuming the OPQOL total score as the dependent variable. All the variables which were significantly associated with QOL in a linear way at the univariate analyses previously described, were included as covariates in the first multivariate model. We chose to include in the model the nominal variable "depression" and not also the "GDS score" because the latter was not available for all participants as previously explained. The second multivariate model considered the variables significantly associated with the OPQOL total score in the first model and included the three SOF criteria instead of the nominal variable "frailty", the six single BADLs instead of the BADL score and the eight single IADLs instead of the IADL score: all these nominal variables were found to be significantly related to the OPQOL score in a linear way at univariate analysis (data not shown). Both models were adjusted for the age of the participants. In order to justify the entry of the variables in the multivariate models multicollinearity was assessed by examining the tolerance values, which resulted to be reasonably high (low values close to zero indicating multiple correlation with other entered variables) [32]. Secondary analyses - both univariate and multivariate - were performed specifically on the "frail" and "robust" groups according to these same selection criteria. Statistical analyses were performed by means of SPSS 14.0 for Windows.

As far as sample size calculations were concerned, we assumed the mean (SD) OPQOL total score among non frail older outpatients to be similar to that found by Bowling [19] in a community-dwelling population aged 65+ [i.e. 134 (14) out of 175], in which the prevalence of frail subjects according to the SOF criteria is expected to be very low, below 5% [31]. Since our sample consisted of outpatients the expected prevalence of frailty was much higher [33], estimated at more than 30%. It was therefore calculated that with a sample of about 240 participants the study would have obtained an almost 80% statistical power at 5% alpha level to detect a difference in the OPQOL total score of at least 5 points out of 175 in frail subjects compared to the rest of the sample.

## Results

The sample was composed of 239 older people living in the community, mainly females (*n *= 164), with an average (SD) age of 81.5 (6.3) years. The participants lived alone in 107 cases (45%) and had at least one carer, informal and/or formal, in 145 cases (61%). They were affected by an average of 4.3 (SD 1.9) chronic diseases and consumed an average of 5.4 (SD 2.9) drugs a day. They suffered from dementia and depression in 62 (26%) (46 women and 16 men, mean age 81.8 years) and 123 (52%) (94 women and 29 men, mean age 81.6 years) cases respectively.

Sixty-one participants (26%) required the help of the non-health volunteer to fill in the OPQOL questionnaire, half of them (31 out of 61) were suffering from dementia. For all participants it was possibile to define frailty status according to the SOF criteria.

### Dimensions of QOL associated with frailty status

According to the SOF criteria 72 participants (30%) were "robust", 89 (37%) were "pre frail" and 78 (33%) were "frail". Besides the OPQOL total score, a number of characteristics of the older subjects were found to be associated with frailty (Table 1 and Additional file 1). If we consider the different aspects of the QOL, almost all dimensions of QOL as described by OPQOL sub-scores were inversely correlated to frailty (i.e. "health", "independence", "home and neighbourhood", "psychological and emotional well-being", and "leisure, activities and religion") except "social relationships and participation" and "financial circumstances" (Table 1 and Additional file 1).

**Table 1:** Characteristics of participants by frailty status (*n *= 239).

Variables	Robust (*n *= 72)	Pre-Frail (*n *= 89)	Frail (*n *= 78)
	**Mean (SD) or % (*n*)**	**Mean (SD) or % (*n*)**	**Mean (SD) or % (*n*)**
	
**Age (years)**	79.4 (6.2)	81.5 (6.2)	83.5 (5.8)
**Civil status**			
**Unmarried**	5 (4)	21 (19)	9 (7)
**Married**	49 (35)	24 (21)	31 (24)
**Divorced**	4 (3)	6 (5)	4 (3)
**Widowed**	42 (30)	49 (44)	57 (44)
**No caregiver**	49 (35)	46 (41)	23 (18)
**Informal Caregiver: Spouse**	26 (19)	9 (8)	17 (13)
**Informal Caregiver: Child**	19 (14)	36 (32)	41 (32)
**Paid personal assistance**	10 (7)	14 (12)	24 (19)
**Living alone**	36 (26)	55 (49)	41 (32)
**Any hospital admission in the past year**	6 (4)	16 (14)	24 (19)
**BADL score^a^**	5.1 (1.4)	4.7 (1.5)	3.3 (1.8)
**IADL score^b^**	5.3 (2.8)	4.8 (2.8)	2.8 (2.2)
**GDS score^c^**	7.4 (4.8)	12.2 (7.6)	13.3 (7.1)
**CIRS m score^d^**	3.7 (1.6)	4.2 (1.7)	5.0 (2.0)
**Any osteomuscular disease**	39 (28)	57 (51)	77 (60)
**Depression**	31 (22)	58 (51)	64 (50)
**Number of drugs taken**	4.4 (2.7)	5.4 (2.8)	6.4 (2.9)
			
**OPQOL total score^e^**	125.9 (13.2)	115.6 (13.9)	107.4 (12.6)
**Life overall**	14.9 (2.4)	13.0 (2.8)	12.0 (3.2)
**Health**	12.9 (2.6)	10.5 (2.8)	8.2 (2.8)
Social relationships and participation	17.8 (3.2)	17.2 (3.3)	17.2 (3.5)
**Independence, control over life, freedom**	14.2 (2.9)	12.4 (3)	10.7 (2.8)
**Home and neighbourhood**	16.7 (2.2)	15.9 (2.4)	15.3 (1.8)
**Psychological and emotional well-being**	15.1 (2.5)	13.7 (2.9)	12.6 (2.6)
Financial circumstances	13.5 (3.2)	13.1 (3.0)	12.8 (3.3)
**Leisure, activities and religion**	20.8 (3.5)	19.7 (3.1)	18.7 (2.5)

Notes: **Variables in bold **are significant at p < 0.05; Besides quality of life assessment, reported in this table are only the variables significantly related to frailty status; All the variables and their p-values are reported in the Additional file 1.

a) Basic Activities of Daily Living. Score range 0 - 6. Higher scores indicate greater independence.

b) Instrumental Activities of Daily Living. Score range 0-8. Higher scores indicate greater independence.

c) Thirty item - Geriatric Depression Scale. Score range 0 - 30. Higher scores indicate worse depressive status. This variable was analysed only in participants without dementia or suffering from mild dementia: 61 subjects belonging to the "robust" group, 77 subjects to the "pre-frail" group and 65 subjects to the "frail" group.

d) Cumulative Illness Rating Scale morbidity. Scores 0-13. Higher scores indicate higher morbidity.

e) Older People's Quality of Life (OPQOL) questionnaire. Total score range 35-175. Higher scores indicate better quality of life.

### Correlates of QOL according to frailty status

In the total sample several variables, including frailty, were significantly associated with a worse QOL at univariate analyses (Table 2 and Additional file 2). Among all these variables, at multivariate regression analysis (model 1 in Table 3), those associated with a worse QOL were being frail, dependence in BADLs and IADLs and depression. However, including in the model the three SOF criteria for frailty, the six specific BADLs and the eight specific IADL items (model 2 in Table 3), besides depression four variables were independently associated with QOL (adjusted R squared 0.39): only one of the SOF criteria for frailty - the "reduced energy level" criterion - and dependence in two BADLs - transferring and bathing - and in a specific IADL - management of money -.

**Table 2:** Characteristics of participants by OPQOL score tertiles in the total sample, among robust and frail participants.

Variables	OPQOL score Lowest tertile	OPQOL score Intermediate tertile	OPQOL score Highest tertile
**Total Sample (*n *= 239)**	Score: 35-109 (*n *= 80)	Score: 110-122 (*n *= 84)	Score: 123-175 (*n *= 75)
	Mean (SD) or % (*n*)	Mean (SD) or % (*n*)	Mean (SD) or % (*n*)
BADL score^a^	3.6 (1.8)	4.3 (1.8)	5.3 (1.2)
IADLscore^b^	3.3 (2.6)	4.1 (2.9)	5.7 (2.4)
MMSE score^c^	24.7 (5.7)	25.1 (4.6)	26.7 (4.0)
GDS score^d^	15.5 (7.1)	10.5 (6.8)	7.8 (5.2)
Depression	71 (57)	50 (42)	32 (24)
Being frail (SOF criteria)	56 (45)	30 (25)	11 (8)
Any fall in the past year	40 (32)	30 (25)	21 (16)
Number of drugs taken	6.1 (3)	5.3 (2.6)	4.7 (2.9)
			
**Robust participants (*n *= 72)**	Score: 35-120 (*n *= 25)	Score: 121-131 (*n *= 23)	Score: 132-175 (*n *= 24)
	Mean (SD)	Mean (SD)	Mean (SD)
BADL score	4.5 (1.8)	5.2 (1.4)	5.6 (0.7)
IADL score	4.2 (3.4)	5 (2.5)	6.8 (1.5)
MMSE score	23.3 (5)	24.5 (5.0)	28.3 (2.9)
Body Mass Index (Kg/m^2^)	24.6 (3.9)	25 (2.8)	27.5 (4.6)
			
**Frail participants (*n *= 78)**	Score: 35-101 (*n *= 28)	Score: 102-113 (*n *= 25)	Score: 114-175 (*n *= 25)
	Mean (SD)	Mean (SD)	Mean (SD)
Age (years)	81.9 (6.3)	83.8 (5.8)	85.0 (5.0)
GDS score	16.1 (7.8)	14.1 (7.1)	10.0 (5.0)

OPQOL = Older People's Quality of Life questionnaire; SOF = Study of Osteoporotic Fractures.

Notes: Reported in this table are only the variables significantly related to the OPQOL score in a linear way at p < 0.05; All the variables concerning the analysis on the total sample and their p-values are reported in the Additional file 2.

a) Basic Activities of Daily Living. Score range 0 - 6. Higher scores indicate greater independence. All 6 specific BADLs turned out to be related to OPQOL score.

b) Instrumental Activities of Daily Living. Score range 0-8. Higher scores indicate greater independence. All 8 specific IADLs turned out to be related to OPQOL score.

c) Mini Mental State Examination. Score range 0 - 30. Higher scores indicate better cognitive function. Scores are corrected for age and education.

d) Thirty item - Geriatric Depression Scale. Score range 0 - 30. Higher scores indicate worse depressive status. This variable was analysed only in participants without dementia or suffering from mild dementia: 63 subjects belonging to the "lowest tertile" group, 70 subjects to the "intermediate tertile" group and 70 subjects to the "highest tertile" group.

**Table 3:** Multiple regression coefficients for OPQOL score in the total sample (*n *= 239).

Variables	Unstandardized Coefficients (95% CI)	Collinearity Tolerance
**Model 1**		
BADL score	**1.46 (0.09 - 2.82)**	0.43
IADL score	**0.94 (0.04 - 1.83)**	0.39
MMSE score	0.24 (-0.18 to 0.65)	0.63
Depression	**-9.41 (-12.75 to -6.07)**	0.90
Being frail (SOF criteria)	**-6.36 (-10.37 to -2.35)**	0.71
CIRS m score	-0.13 (-1.20 to 0.94)	0.65
Any fall in the past year	-1.84 (-5.38 to 1.68)	0.95
Number of drugs taken	-0.15 (-0.83 to 0.54)	0.65
**Model 2**		
Depression	**-8.05 (-11.36 to -4.74)**	0.83
Dependence in BADLs		
Transferring	**-6.14 (-11.61 to -0.68)**	0.49
Eating	-6.64 (-13.51 to 0.23)	0.66
Bathing	**-5.23 (-10.31 to -0.14)**	0.36
Dressing	2.86 (-2.61 to 8.33)	0.39
Toileting	3.03 (-2.96 to 9.02)	0.48
Continence	-2,36 (-5.89 to 1.17)	0.73
Dependence in IADLs		
Using the telephone	-0.80 (-6.53 to 4.94)	0.60
Shopping	-1.19 (-6.18 to 3.81)	0.38
Doing housework	5.17 (-1.02 to 11.35)	0.25
Preparing meals	-4.43 (-10.37 to 1.51)	0.26
Doing laundry	-0.96 (-7.45 to 5.52)	0.22
Taking medications	3.35 (-1.32 to 8.03)	0.44
Managing transportation	-1.46 (-6.58 to 3.66)	0.35
Money management	**-8.37 (-12.99 to -3.75)**	0.46
Frailty (SOF criteria)		
Weight loss	-4.19 (-9.21 to 0.83)	0.90
Rising from a chair 5 times	-1.44 (-5.81 to 2.94)	0.50
Reduced energy level	**-6.72 (-10.21 to -3.23)**	0.76

Notes: **Bold values **are significant at p < 0.05; Both models are adjusted for age; Model 1 adjusted R squared = 0.32; Model 2 adjusted R squared = 0.39.

OPQOL = Older People's Quality of Life questionnaire; BADLs = Basic Activities of Daily Living; IADLs = Instrumental Activities of Daily Living; MMSE = Mini Mental State Examination; SOF = Study of Osteoporotic Fractures; CIRS m = Cumulative Illness Rating Scale morbidity.

The secondary analyses showed that among "frail" participants both a better emotional status and a more advanced age were associated with a better QOL (Table 2 and Table 4), whereas among "robust" participants only the BMI was directly associated with QOL (Table 2 and Table 4).

**Table 4:** Multiple regression coefficients for OPQOL score among robust and frail participants.

Variables	Unstandardized Coefficient (95% CI)	Collinearity Tolerance
**Robust participants (*n *= 72)**		
BADL score	1.90 (-0.90 to 4.69)	0.48
IADL score	0.64 (-1.13 to 2.40)	0.31
MMSE score	0.34 (-0.52 to 1.19)	0.45
Body Mass Index (Kg/m^2^)	**0.89 (0.17 - 1.60)**	0.95
**Frail participants (*n *= 78)**		
**Model without GDS score (*n *= 78)**		
Age (years)	**0.49 (0.01 - 0.99)**	0.97
Depression	-0.84 (-6.76 to 5.09)	0.97
**Model with GDS score (*n *= 65)**		
Age (years)	**0.55 (0.02 - 1.08)**	0.96
GDS score	**-0.50 (-0.91 to -0.09)**	0.96

Notes: **Bold values **are significant at p < 0.05; Models are adjusted for age; "Robust participants" model adjusted R squared = 0.22; "Frail participants" model (with GDS score) adjusted R squared = 0.15.

OPQOL = Older People's Quality of Life questionnaire; BADLs = Basic Activities of Daily Living; IADLs = Instrumental Activities of Daily Living; MMSE = Mini Mental State Examination; GDS = Geriatric Depression Scale.

## Discussion

In this cross-sectional study of the complex relationship between frailty status and generic QOL in a sample of community-dwelling older outpatients without severe dementia, we used two recently validated assessment tools: the SOF criteria for frailty status, which were demonstrated to be applicable to the whole sample, and the OPQOL. The OPQOL has excellent applicability to cognitively normal subjects [19], and was shown here to be applicable to people suffering from mild to moderate dementia. The CGA we used included several important social factors determining health in older age, such as recent life events, housing, financial status and social isolation [34]. The high prevalence of frailty, dementia and depression that we found in the sample could be accounted for by the specific setting of the study which involved geriatric outpatients. This hypothesis is supported by the fact that in another recent study on older outpatients with a disability referred to the same geriatric service the prevalence of depressive disorders was found to be even greater (i.e. over 70%) [35].

### Dimensions of QOL associated with frailty status

As far as the correlates of frailty were concerned, consistent with other studies we found that frail subjects reported a worse overall QOL than pre-frail and non-frail subjects [3,8,10-14,36]. Moreover, according to the findings of this study as many as five of the seven dimensions of QOL that we investigated were found to be impaired in frail older participants. This suggests that interventions targeting QOL in frail community-dwelling older outpatients should consider as outcomes, not only health-related QOL, but also other domains of QOL, such as functional independence, psychological well-being, home and neighbourhood, leisure activities and religion. Only the QOL domains of "social relationships and participation" and "financial circumstances" were not significantly different among the three "frailty status" groups.

These findings are consistent with i) the objective variables which were associated with frailty, such as functional dependence, depression and comorbidity, already highlighted by recent studies [3,10-12,37], ii) the fact that "frail" participants had higher levels of formal and informal personal support, and iii) the fact that living and financial conditions were similar along the three groups. As far as the latter point is concerned, it is worth noting that the study not only considered family income but also housing tenure which, along with housing value, has been shown to be highly correlated with socioeconomic status in older people [38]. Recent studies have demonstrated that socioeconomic factors have a greater influence on physical disability at younger than older ages [39] and that among older adults aged 65-74 the association between social inequalities and frailty appears to be mediated by comorbidity [37]. However, even in older subjects socioeconomic inequalities could be responsible for developing functional impairment and certain illnesses [40]. We cannot therefore exclude that, in studying a sample of outpatients, we might have selected a group of community-dwelling older adults with better social and health assistance for whom possible differences in socioeconomic status may have no impact on frailty.

### Correlates of QOL according to frailty status

The clinical and functional characteristics independently associated with a worse QOL were: frailty, but with only one of the three SOF criteria being involved, i.e. "reduced energy level"; disability in the "transferring" and "bathing" BADLs and in the "management of money" IADL; depressive status, consistently with available evidence [7,41,42]. A possible explanation for the "reduced energy level" SOF item could be an increased production of specific cytokines such as TNF α [15], which has already been postulated in the pathophysiology of frailty [43] and could account for the development of a constellation of non-specific symptoms such as weakness, malaise and fatigue [44]; these could in turn explain a deterioration in QOL. Moreover, closely related to the concept of a "reduced energy level" is that of *anergia*, namely self-reported lack of energy, which has been shown to be associated with a poorer life satisfaction and a higher mortality risk [45].

With regard to the relationship between functional status and QOL, Bowling and colleagues reported that *perceived *self-efficacy discriminated between perceived QOL as "good", or "not good", among people aged 65+ with severe disabilities [46]. The IADL index captures disability at an earlier stage of the disabling process than the BADL index [47], when the psychological processes of adaptation to disability - discussed in the following paragraph - are not yet fully developed. The management of money is only one of the skills which are lost early in the disabling process [48,49], but it could have a greater impact on QOL than the loss of other IADLs. This might be because it implies that older people with mild mental impairment *perceive *less control over their lives since they depend on others in the use of their own money. The relationship between the transferring and bathing BADL abilities and QOL that we found in this study confirms the well-known relevance of limitations in balance, mobility and self-efficacy in affecting QOL [50,51]. Objective indicators of wealth were not related to QOL not only in our sample but also in other studies, possibly because in older age, when incomes are more levelled, these indicators are less sensitive than subjectively perceived financial circumstances [6,52].

In "frail" older subjects, a better emotional status and a more advanced age were directly associated with QOL. The association with age suggests that it takes time for an adaptive response to the *frailty identity crisis *[15] to occur; this has already been observed in the adaptation to comorbidity and disability by means of the *response shift phenomenon *[44,53,54], a term which has been coined to describe the way the psychological and practical compensatory actions following physical deterioration account for a lack of change in the perceived QOL [46,53]. In this perspective our findings support the need for research on interventions that address psychological and emotional well-being to improve QOL among frail older adults.

Among "robust" older subjects, the only independent predictor of QOL was the BMI. The association between a higher BMI and a better QOL is supported by recent studies demonstrating that the optimal BMI for the maintenance of functional capacity in older people may be above the normal limit [55], i.e. between 23 and 30 Kg/m^2 ^[56,57]. Thus, in robust older people a BMI within this range might also promote a better QOL.

### Limitations of the study

Since the sample considered in this study consisted of outpatients, our findings cannot be extended to the entire population of older people living at home. However, it must be noted that frail subjects make larger use of health and community services than subjects who are not frail [33]. Thus, the findings of this study may be useful to promote QOL in the frail elders referred to outpatient services in the community.

The cross-sectional design of the study did not allow us to consider temporary trajectories of QOL. A recent research by Solomon and colleagues showed that individual ratings of QOL are highly variable over time in community-dwelling elderly people with advanced illness (cancer, heart failure and chronic obstructive pulmonary disease) and that declining QOL is not an inevitable consequence of advancing illness [42]. Interestingly, even in this longitudinal study, which did not consider frailty among the covariates, functional status and depression turned out to be determinants of QOL [42].

Another limitation of our study was the limited size of the subgroups of "frail" and "robust" subjects on which the secondary statistical analyses were performed. These preliminary findings from the secondary analyses will therefore have to be confirmed by longitudinal studies carried out on larger populations of older adults living in the community.

## Conclusions

In sum, five of the seven dimensions of QOL were negatively affected by frailty, but only one SOF criterion for frailty (reduced energy level) was independently related to QOL after correction for age, functional status and depression. Correlates of a better QOL were a more advanced age and a better emotional status for frail elders, and a higher BMI for robust older people. Interventions targeting the QOL in frail community-dwelling older outpatients should consider as outcomes, not only health-related QOL, but also other domains of the QOL.

## Competing interests

The authors declare that they have no competing interests.

## Authors' contributions

CB was responsible for the data, contributed to the literature review, study design, statistical analyses and drafted the manuscript. AB contributed to the literature review, statistical analyses and discussion section. AC, PN, SM and MC were involved in data collection. CV was responsible for the data and contributed to the literature review and discussion section. All authors have read and approved the final manuscript.

## Supplementary Material

Additional file 1 **Characteristics of participants by frailty status**. The data provided represent the characteristics of the 239 participants according to frailty status. Click here for file

Additional file 2 **Characteristics of participants by OPQOL score tertiles**. The data provided represent the characteristics of the 239 participants according to OPQOL score tertiles. Click here for file
