# Supplementary material for: Dimensions and correlates of quality of life according to frailty status: a cross-sectional study on community-dwelling older adults referred to an outpatient geriatric service in Italy
Source: Health Qual Life Outcomes. 2010 Jun 8;8:56. doi: 10.1186/1477-7525-8-56 (PMC2889875; doi:10.1186/1477-7525-8-56)
Supplement: Additional file 1 — Characteristics of participants by frailty status. The data provided represent the characteristics of the 239 participants according to frailty status. [file 1477-7525-8-56-S1.DOC]

**Additional file 1**

Characteristics of participants by frailty status.

| Variables | Robust  (*n* = 72) | Pre-Frail  (*n* = 89) | Frail  (*n* = 78) |  |
| --- | --- | --- | --- | --- |
|  | Mean (SD) or % (*n*) | Mean (SD) or % (*n*) | Mean (SD) or % (*n*) | P |
| **Demographic characteristics** |  |  |  |  |
| Age (years) | 79.4 (6.2) | 81.5 (6.2) | 83.5 (5.8) | < 0.001** |
| Sex: female | 58 (42) | 71 (63) | 76 (59) | 0.063 |
| Education |  |  |  | 0.768 |
| None or primary school | 64 (46) | 70 (62) | 77 (60) |  |
| Secondary school | 31 (22) | 25 (22) | 18 (14) |  |
| University | 5 (4) | 5 (5) | 5 (4) |  |
| Civil status |  |  |  | 0.004* |
| Unmarried | 5 (4) | 21 (19) | 9 (7) |  |
| Married | 49 (35) | 24 (21) | 31 (24) |  |
| Divorced | 4 (3) | 6 (5) | 4 (3) |  |
| Widowed | 42 (30) | 49 (44) | 57 (44) |  |
| **Care provision** |  |  |  |  |
| No caregiver | 49 (35) | 46 (41) | 23 (18) | 0.002* |
| Informal Caregiver | 46 (33) | 47 (42) | 62 (48) | 0.094 |
| Spouse | 26 (19) | 9 (8) | 17 (13) | 0.013* |
| Children | 19 (14) | 36 (32) | 41 (32) | 0.013* |
| Other | 0 (0) | 2 (2) | 4 (3) | 0.327 † |
| Age (years) | 64.6 (14.2) | 56.5 (12.4) | 60.7 (15.8) | 0.336 |
| Sex: female | 76 (25) | 71 (30) | 73 (35) | 0.914 |
| Paid personal assistance | 10 (7) | 14 (12) | 24 (19) | 0.037* |
| Age (years) | 36.7 (7.2) | 42.2 (11.8) | 43.7 (10.8) | 0.171 |
| Sex: female | 100 (7) | 83 (10) | 100 (19) | 0.102 |
| Daily hours of assistance | 14.9 (11.4) | 14.8 (10.2) | 13.8 (10.7) | 0.773 |
| **Living and financial conditions** |  |  |  |  |
| Living alone | 36 (26) | 55 (49) | 41 (32) | 0.040* |
| Home ownership | 81 (58) | 72 (64) | 68 (53) | 0.206 |
| Home surface area (sq. meters) | 87.4 (42.6) | 73.2 (29.2) | 80.8 (36.6) | 0.289 |
| Yearly family income |  |  |  | 0.323 |
| < 10,000 euro | 14 (10) | 18 (16) | 17 (13) |  |
| 10,000-20,000 euro | 51 (37) | 64 (57) | 58 (45) |  |
| 20,000-30,000 euro | 19 (14) | 11 (10) | 18 (14) |  |
| > 30,000 euro | 15 (11) | 7 (6) | 8 (6) |  |
| **Life events in the past year** |  |  |  |  |
| Any life event | 49 (35) | 52 (46) | 62 (48) | 0.244 |
| Any fall | 25 (18) | 28 (25) | 39 (30) | 0.165 |
| ED admission | 25 (18) | 29 (26) | 41 (32) | 0.088 |
| Hospital admission | 6 (4) | 16 (14) | 24 (19) | 0.006* |
| Any severe acute disease | 4 (3) | 3 (3) | 10 (8) | 0.180 † |
| Bereavement | 4 (3) | 5 (4) | 0 (0) | 0.159 † |
| Being victim of crime | 1 (1) | 1 (1) | 0 (0) | 0.756 † |
| **Physical and Functional status** |  |  |  |  |
| Body Mass Index (Kg/m^2^) | 25.7 (4.0) | 26.1 (4.1) | 25.7 (5.4) | 0.994 |
| BADL score ^a^ | 5.1 (1.4) | 4.7 (1.5) | 3.3 (1.8) | < 0.001** |
| IADL score ^b^ | 5.3 (2.8) | 4.8 (2.8) | 2.8 (2.2) | < 0.001** |
| MMSE score ^c^ | 25.4 (4.9) | 26.0 (4.6) | 25 (5.2) | 0.641 |
| GDS score ^d^ | 7.4 (4.8) | 12.2 (7.6) | 13.3 (7.1) | < 0.001** |
| **Comorbidity** |  |  |  |  |
| CIRS m score ^e^ | 3.7 (1.6) | 4.2 (1.7) | 5.0 (2.0) | < 0.001** |
| Any osteomuscular disease | 39 (28) | 57 (51) | 77 (60) | < 0.001* |
| Dementia | 28 (20) | 21 (19) | 30 (23) | 0.446 |
| Depression | 31 (22) | 58 (51) | 64 (50) | < 0.001* |
| Number of drugs taken | 4.4 (2.7) | 5.4 (2.8) | 6.4 (2.9) | < 0.001** |
| **Quality of life assessment** |  |  |  |  |
| OPQOL total score ^f^ | 125.9 (13.2) | 115.6 (13.9) | 107.4 (12.6) | < 0.001** |
| Life overall | 14.9 (2.4) | 13.0 (2.8) | 12.0 (3.2) | < 0.001** |
| Health | 12.9 (2.6) | 10.5 (2.8) | 8.2 (2.8) | < 0.001** |
| Social relationships, participation | 17.8 (3.2) | 17.2 (3.3) | 17.2 (3.5) | 0.271 |
| Independence, control over life | 14.2 (2.9) | 12.4 (3) | 10.7 (2.8) | < 0.001** |
| Home and neighbourhood | 16.7 (2.2) | 15.9 (2.4) | 15.3 (1.8) | < 0.001** |
| Psychological well-being | 15.1 (2.5) | 13.7 (2.9) | 12.6 (2.6) | < 0.001** |
| Financial circumstances | 13.5 (3.2) | 13.1 (3.0) | 12.8 (3.3) | 0.157 |
| Leisure, activities and religion | 20.8 (3.5) | 19.7 (3.1) | 18.7 (2.5) | < 0.001** |

* P < 0.05 at Pearson’s chi-squared test; ** P < 0.05 at one-way ANOVA and P > 0.05 at the test for departure from the linear trend; † statistical analysis performed by means of Fisher’s exact test.

ED = emergency department.

a) Basic Activities of Daily Living. Score range 0 – 6. Higher scores indicate greater independence.

b) Instrumental Activities of Daily Living. Score range 0-8. Higher scores indicate greater independence.

c) Mini Mental State Examination. Score range 0 – 30. Higher scores indicate better cognitive function. Scores are corrected for age and education.

d) Thirty item – Geriatric Depression Scale. Score range 0 – 30. Higher scores indicate worse depressive status. This variable was analysed only in participants without dementia or suffering from mild dementia: 61 subjects belonging to the “robust” group, 77 subjects to the “pre frail” group and 65 subjects to the “frail” group.

e) Cumulative Illness Rating Scale morbidity. Scores 0-13. Higher scores indicate higher morbidity.

f) Older People’s Quality of Life (OPQOL) questionnaire. Total score range 35-175. Higher scores indicate better quality of life.
