# Supplementary material for: Dimensions and correlates of quality of life according to frailty status: a cross-sectional study on community-dwelling older adults referred to an outpatient geriatric service in Italy
Source: Health Qual Life Outcomes. 2010 Jun 8;8:56. doi: 10.1186/1477-7525-8-56 (PMC2889875; doi:10.1186/1477-7525-8-56)
Supplement: Additional file 2 — Characteristics of participants by OPQOL score tertiles. The data provided represent the characteristics of the 239 participants according to OPQOL score tertiles. [file 1477-7525-8-56-S2.DOC]

**Additional file 2**

Characteristics of participants by OPQOL score tertiles.

| Variables | Lowest tertile  OPQOL score:  35-109  (*n* = 80) | Intermediate tertile  OPQOL score:  110-122  (*n* = 84) | Highest tertile  OPQOL score:  123-175  (*n* = 75) |  |
| --- | --- | --- | --- | --- |
|  | Mean (SD) or % (*n*) | Mean (SD) or % (*n*) | Mean (SD) or % (*n*) | P |
| **Demographic characteristics** |  |  |  |  |
| Age (years) | 82.0 (6.1) | 81.9 (6.8) | 80.6 (5.8) | 0.162 |
| Sex: female | 73 (58) | 66 (55) | 68 (51) | 0.619 |
| Education |  |  |  | 0.082 |
| None or primary school | 68 (55) | 78 (66) | 63 (47) |  |
| Secondary school | 27 (21) | 18 (15) | 29 (22) |  |
| University | 5 (4) | 4 (3) | 8 (6) |  |
| Civil status |  |  |  | 0.137 |
| Unmarried | 22 (17) | 10 (8) | 7 (5) |  |
| Married | 28 (22) | 35 (29) | 39 (29) |  |
| Divorced | 6 (5) | 4 (3) | 4 (3) |  |
| Widowed | 46 (36) | 52 (44) | 51 (38) |  |
| **Care provision** |  |  |  |  |
| No caregiver | 34 (27) | 39 (33) | 45 (34) | 0.337 |
| Informal Caregiver | 55 (44) | 51 (43) | 48 (36) | 0.683 |
| Spouse | 14 (11) | 19 (16) | 17 (13) | 0.653 |
| Children | 38 (30) | 30 (25) | 31 (23) | 0.520 |
| Other | 4 (3) | 2 (2) | 0 (0) | 0.328 † |
| Age (years) | 58.9 (16.3) | 61.6 (13.2) | 60.6 (13.9) | 0.573 |
| Sex: female | 80 (35) | 72 (31) | 67 (24) | 0.425 |
| Paid personal assistance | 18 (14) | 19 (16) | 11 (8) | 0.315 |
| Age (years) | 35.3 (9.7) | 46.6 (9.4) | 44.3 (9.5) | 0.015 |
| Sex: female | 100 (14) | 94 (15) | 88 (7) | 0.438 |
| Daily hours of assistance | 11.5 (10.7) | 16.3 (10.3) | 15.1 (10.1) | 0.320 |
| **Living and financial conditions** |  |  |  |  |
| Living alone | 48 (38) | 39 (33) | 48 (36) | 0.454 |
| Home ownership | 66 (53) | 75 (63) | 79 (59) | 0.197 |
| Home surface area (sq. meters) | 76.4 (36.9) | 76.8 (30.8) | 87.2 (40.8) | 0.069 |
| Yearly family income |  |  |  | 0.675 |
| < 10,000 euro | 18 (14) | 13 (11) | 19 (14) |  |
| 10,000-20,000 euro | 60 (48) | 63 (53) | 51 (38) |  |
| 20,000-30,000 euro | 14 (11) | 17 (14) | 17 (13) |  |
| > 30,000 euro | 9 (7) | 7 (6) | 13 (10) |  |
| **Life events in the past year** |  |  |  |  |
| Any life event | 63 (50) | 52 (44) | 47 (35) | 0.133 |
| Any fall | 40 (32) | 30 (25) | 21 (16) | 0.041* |
| ED admission | 40 (32) | 32 (27) | 23 (17) | 0.068 |
| Hospital admission | 20 (16) | 17 (14) | 9 (7) | 0.173 |
| Any severe acute disease | 5 (4) | 5 (4) | 8 (6) | 0.656 † |
| Bereavement | 3 (2) | 4 (3) | 3 (2) | 0.999 † |
| Being victim of crime | 1 (1) | 1 (1) | 0 (0) | 0.999 † |
| **Physical and functional status** |  |  |  |  |
| Body Mass Index (Kg/m^2^) | 25.6 (4.9) | 25.9 (4.6) | 26.2 (3.9) | 0.443 |
| BADL score ^a^ | 3.6 (1.8) | 4.3 (1.8) | 5.3 (1.2) | < 0.001** |
| IADL score ^b^ | 3.3 (2.6) | 4.1 (2.9) | 5.7 (2.4) | < 0.001** |
| MMSE score ^c^ | 24.7 (5.7) | 25.1 (4.6) | 26.7 (4.0) | 0.013** |
| GDS score ^d^ | 15.5 (7.1) | 10.5 (6.8) | 7.8 (5.2) | < 0.001** |
| Being frail (SOF criteria) | 56 (45) | 30 (25) | 11 (8) | < 0.001* |
| **Comorbidity** |  |  |  |  |
| CIRS m score ^e^ | 4.6 (1.8) | 4.3 (1.9) | 4.1 (1.8) | 0.087 |
| Any osteomuscular disease | 70 (56) | 50 (42) | 55 (41) | 0.026* |
| Dementia | 30 (24) | 32 (27) | 15 (11) | 0.026* |
| Depression | 71 (57) | 50 (42) | 32 (24) | < 0.001* |
| Number of drugs taken | 6.1 (3) | 5.3 (2.6) | 4.7 (2.9) | 0.002** |

* P < 0.05 at Pearson’s chi-squared test; ** P < 0.05 at one-way ANOVA and P > 0.05 at the test for departure from the linear trend; † statistical analysis performed by means of Fisher’s exact test.

OPQOL = Older People’s Quality of Life questionnaire; ED = emergency department; SOF = Study of Osteoporotic Fractures.

a) Basic Activities of Daily Living. Score range 0 – 6. Higher scores indicate higher independence.

b) Instrumental Activities of Daily Living. Score range 0-8. Higher scores indicate higher independence.

c) Mini Mental State Examination. Score range 0 – 30. Higher scores indicate better cognitive function. Scores are corrected for age and education.

d) Thirty item – Geriatric Depression Scale. Score range 0 – 30. Higher scores indicate worse depressive status. This variable was analysed only in participants without dementia or suffering from mild dementia: 63 subjects belonging to the “lowest tertile” group, 70 subjects to the “intermediate tertile” group and 70 subjects to the “highest tertile” group.

e) Cumulative Illness Rating Scale morbidity. Scores 0-13. Higher scores indicate higher morbidity.
